# Diagnostic approach to dementia in Latin America

**DOI:** 10.1002/alz70858_101765

**Published:** 2025-12-24

**Authors:** Sylvia E. Josephy‐Hernandez

**Affiliations:** ^1^ Universidad de Costa Rica, San Jose, San Jose, Costa Rica; Hospital Mexico, Caja Costarricense de Seguro Social, San Jose, San Jose, Costa Rica

## Abstract

Obtaining a diagnosis is key for patient autonomy and care planning. Accuracy in the diagnosis allows for tailored therapies and participation in clinical research. Likewise, a specific dementia diagnosis allows for better understanding of the symptoms and progression observed by patients and caregivers. Diagnosing a specific type of dementia in Latin America requires adapting guidelines to the available ancillary diagnostic tools.

In the diagnostic formulation proposed by Dickerson, et al., there are three diagnostic tiers: 1. Cognitive Functional Status, 2. Cognitive‐Behavioral Syndrome, and 3. Neurodegenerative Brain Disease.

The cognitive functional status refers to how independent the person is, ranging from cognitively normal, to severe dementia. Our experience in Latin America is that families often compensate for patients’ deficits and will most often minimize their level of impairment. This requires a careful understanding of the patient's baseline and current abilities.

Defining the cognitive‐behavioral syndrome refers to the clinical type of dementia. For example, amnestic multidomain dementia, behavioral variant frontotemporal dementia, posterior cortical atrophy, etc. We use the symptoms described by the patient and informants, in addition to the signs obtained through bedside and formal neuropsychological evaluations and the neurologic exam.

Lastly, the neurodegenerative brain disease refers to the suspected underlying pathology (Alzheimer's disease, frontotemporal lobar degeneration, etc.). This information requires ancillary testing which will vary widely depending on the practice setting. Imaging techniques include both structural (CT, MRI) and functional (PET, SPECT). The underlying brain pathology may be specified in clinical practice with genetic testing and biomarkers. The latter includes fluid (blood‐based and CSF), tissue (skin), and imaging (PET). One may use many combinations of the listed tools to reach the most precise diagnosis.

In this presentation, we will provide a guide on best practices with an adaptation to the Latin American culture and combination of available resources.

